# Association of serum docosahexaenoic acid and eicosapentaenoic acid levels with dietary intakes and supplement use during pregnancy: a prospective observational study

**DOI:** 10.1017/jns.2023.105

**Published:** 2023-12-14

**Authors:** Nana Wakabayashi, Megumi Haruna, Kaori Yonezawa, Emi Sasagawa, Yuriko Usui, Riko Ohori, Satoko Aoyama, Satoshi Sasaki, Takeshi Nagamatsu, Tokyo area members of the J-PEACH Study group as of 2019–2022

**Affiliations:** 1 Department of Midwifery and Women's Health, Graduate School of Medicine, The University of Tokyo, Tokyo, Japan; 2 Department of Social and Preventive Epidemiology, School of Public Health Division of Health Sciences and Nursing, Graduate School of Medicine, The University of Tokyo, Tokyo, Japan; 3 Department of Obstetrics and Gynecology, Facility of Medicine, The University of Tokyo, Tokyo, Japan

**Keywords:** Docosahexaenoic acid, Eicosapentaenoic acid, Maternal diet, Pregnancy, Supplementation

## Abstract

This study aimed to determine the association of serum docosahexaenoic acid (DHA) and eicosapentaenoic acid (EPA) levels with dietary intakes and supplement use during pregnancy. This prospective observational study was conducted at a university hospital in Tokyo, Japan. Participants in their second and third trimesters were given a self-administered questionnaire assessing the frequency of DHA and EPA supplement use in the past month and a brief-type self-administered diet history questionnaire. Non-fasting serum DHA and EPA levels were analysed using gas chromatography. Differences in biomarkers by frequency of supplement use were determined using multiple comparison analyses, and Spearman's correlation coefficient was used to determine biomarkers and DHA and EPA intakes by food group. Of the 116 participants, 11 (9⋅5 %) in the second trimester and 18 (15⋅5 %) in the third trimester regularly used supplements (≥5 times per week). Regular users had higher serum DHA and EPA levels than never users in the second and third trimesters. Dietary DHA and EPA intake from fish and shellfish was positively correlated with serum DHA and EPA in the second and third trimesters. Supplement use ≥5 times per week and fish and shellfish intake were associated with high serum DHA and EPA levels.

## Graphical abstract

**Figure:**
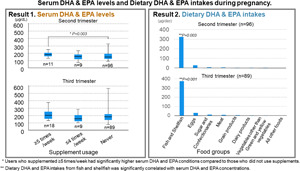

## Introduction

Fatty acids, which are a constituent of cell membranes and influence several hormonal functions, are important nutrients for pregnant women. Previous studies have shown that high levels of maternal docosahexaenoic acid (DHA) and/or eicosapentaenoic acid (EPA) are related to a reduction of maternal depressive symptoms.^(1–4)^ Additionally, some studies have shown that higher maternal blood DHA and EPA levels prevent preterm birth.^(5–8)^

Circulating maternal DHA and EPA levels are influenced by both food intake and supplement uses, as well as maternal age, educational level, economic status, smoking, and alcohol consumption.^(9–12)^ Previous studies have shown that higher intakes of DHA- and EPA-containing foods, particularly fish and shellfish, result in higher maternal serum DHA and EPA levels.^(11,12)^ An interventional study has shown that supplements containing DHA and/or EPA increase maternal serum DHA and EPA levels.^(13)^

According to a Japanese study, the percentage of pregnant women who use DHA and/or EPA supplements is 2⋅57 %.^(1)^ To our knowledge, no studies in Japan have reported the influence of DHA and/or EPA supplement use on maternal serum DHA and EPA levels. Further, there is a lack of information on the types of diets to increase DHA and EPA levels in pregnant women in Japan. Some pregnant women in Japan might restrict the amount of consumption of seafood. Although adults in Japan are known to have the highest dietary intake of *n*-3 fatty acids worldwide,^(6,14,15)^ the National Health and Nutrition Survey in Japan has shown that seafood intake has decreased from 1995 to 2019.^(16)^ Moreover, the Ministry of Health, Labour, and Welfare in Japan has warned pregnant women about consuming large quantities of seafood due to its high mercury content.^(17)^

It is important to consider not only food intake or supplement use but also hormonal changes during pregnancy. Some studies have shown an increase in absolute maternal serum DHA and EPA levels from early to late pregnancy.^(11,18)^ This is thought to reflect the increased fetal demand for DHA and EPA towards the later stages of pregnancy.^(19,20)^ Additionally, maternal blood may store more DHA and EPA from dietary intake or supplement use, particularly in the later stages of pregnancy.

This study aimed to determine the relationship between the usage of supplements and maternal blood DHA and EPA concentrations in the second and third trimesters of pregnancy and also to find out the current DHA and EPA-containing foods in Japan that are related to maternal blood DHA and EPA levels in the absence of supplement use in the second and third trimesters.

## Experimental methods

### Study design and participants

This prospective observational study was part of a cohort study.^(21)^ The participants included pregnant women attending the obstetric outpatient department of a tertiary emergency medical facility in Tokyo, Japan, from March 2020 to July 2021. The inclusion criteria were as follows: age ≥18 years and the ability to read and write in Japanese. The exclusion criteria were as follows: planned delivery in another facility or assessed as unable to participate in the study by clinical staff. All the women who participated in this study provided written informed consent at the time of recruitment. An e-mail with the access link to the second-trimester questionnaire was sent to the participants during weeks 18–21 of their pregnancies with a request to complete it by 27 weeks and 6 d. If participants had not completed the questionnaire within 2 weeks of receiving it, a reminder e-mail was sent. An e-mail with the link to access the third-trimester questionnaire was sent during week 35 of their pregnancies, with a request to complete it by 38 weeks and 6 d. If participants had not completed the questionnaire within 1 week of receiving it, a reminder e-mail was sent.

This study was conducted according to the guidelines laid down in the Declaration of Helsinki and all procedures involving patients were approved by the Ethics Committee of the University of Tokyo (approval no.: 2019318NI). Written informed consent was obtained from all patients.

### Participant characteristics

Maternal age at delivery, pre-pregnancy body mass index (BMI), marital status, educational level, annual family income, employment status, parity, alcohol habits, and information about supplement use were obtained from the questionnaires or the participants’ medical records after delivery.

### Blood sampling and measurement

Non-fasting blood samples were collected from participants during routine perinatal check-ups in the second trimester (17–22 gestational weeks) and the third trimester (34–39 gestational weeks). Whole blood was collected in serum separator tubes (Terumo, VENOJECT2). After collection, the serum was separated by centrifugation at 3500 rpm for 5 min at room temperature and stored at −80 °C. Samples were assessed within 17 months of collection.

The blood samples were assayed for DHA and EPA. Biomarkers were measured using a contract laboratory (SRL, Inc., Tokyo, Japan). The samples were analysed by gas chromatography using a GC-2010 (Shimadzu Corporation, Kyoto, Japan) with a TC-70 capillary column (GL Sciences Inc., Tokyo, Japan). The data are expressed as absolute values (μg/mL).

### Dietary intake

The brief-type self-administered diet history questionnaire (BDHQ), which was validated for the measurement of DHA- and EPA-containing intakes among pregnant women in Japan,^(22)^ was used to collect data on the participants’ intakes of various foods in the previous month. Dietary intake estimates of 58 foods and beverages and their energy and selected nutrient contents were calculated using an *ad hoc* computer algorithm developed for BDHQ, based on the Standard Tables of Food Composition in Japan.^(23)^ We calculated DHA and EPA intakes from each food group. In the analyses, DHA and EPA intakes were energy-adjusted using the density method (%/4184000 J) to reduce inter-individual measurement errors. The *n*-3 fatty acid intakes from each food group included the intakes of other fatty acids that are similar to DHA and EPA, e.g., alpha-linolenic acid. The data of participants who reported extremely low energy intakes (<2510400 J/d) were excluded before performing the statistical analysis. None of the participants reported an extremely high energy intake (≥16736000 J/d).

### Supplement use

The previous month's usage of DHA and/or EPA supplements was obtained from the questionnaire for both the second and third trimesters. Participants were asked *ad hoc* questions, ‘Do you use an *n*-3 fatty acid (DHA and/or EPA) supplement?’ and ‘How often do you use it (every day, 5–6 times/week, 3–4 times/week, 1–2 times/week, sometimes, or never)?’ Based on a previous study,^(24)^ regular use, irregular use, and never use were defined as ≥5 times per week, ≤4 times per week, and never, respectively.

### Statistical analysis

Pregnant women who completed both questionnaires and had biomarker data for both the second and third trimesters were included in the analyses.

Categorical variables are presented as frequencies and percentages. Continuous variables are presented as means ± standard deviations (sd). The paired Student's *t*-test was used to investigate differences in dietary DHA and EPA intakes (%/4184000 J) and serum DHA and EPA levels between the second and third trimesters.

The analysis aimed to determine the association between the use of DHA and/or EPA supplementation and serum DHA and EPA levels. Medians and interquartile ranges (IQRs) are used to describe the three frequencies of supplement use groups: regular (every day, 5–6 days/week), irregular (3–4 days/week, 1–2 days/week, ≤1 d/week), and never. A Tukey's multiple comparison analysis was performed to investigate the differences in serum DHA and EPA levels and dietary intakes among the three groups.

The analysis also aimed to determine the association between the dietary intakes of DHA and EPA by food group (mg/d) and serum DHA and EPA levels using Spearman's rank correlation coefficient. This analysis was conducted among non-users of DHA and/or EPA supplements during the second (*n* 96) and third (*n* 89) trimesters to eliminate the effects of supplementation. All analyses were performed using SPSS version 27⋅0 for Microsoft Windows (IBM Corp., Armonk, NY).

## Results

### Participants

**Fig. 1.**
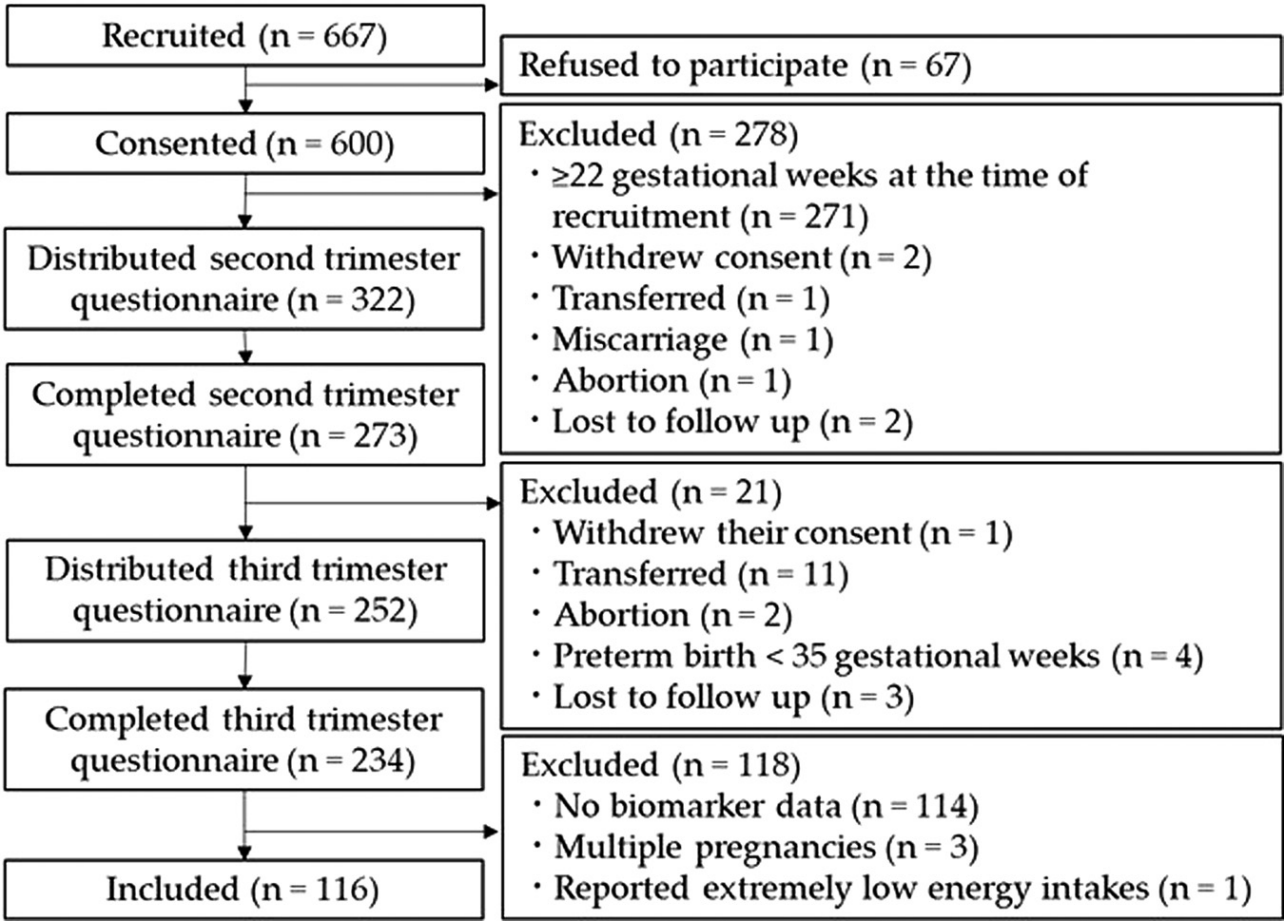
Flow chart of the study.

Of the 600 women who consented to participate in the study, 234 completed the questionnaires during their second and third trimesters (Fig. 1). After excluding the data of participants who had no biomarker data (*n* 114), multiple pregnancies (*n* 3), or extremely low energy intakes (<2510400 J/d) (*n* 1), 116 women were included in the study.

### Participant characteristics

Table 1 shows the participant characteristics. The mean ± sd maternal age at the time of delivery was 35⋅3 ± 4⋅3 years. Of the participants, 77⋅6 % had graduated from university, and 72⋅5 % had an annual family income of over 7 million Japanese yen. Although nine participants (7⋅8 %) had been smoking pre-pregnancy, none smoked at the time of the study. In addition, none of the participants had consumed alcohol in the previous month. In the second and third trimesters, eleven (9⋅5 %) and eighteen (15⋅5 %) women used supplements ≥5 times per week, respectively. The mean dietary DHA and EPA intakes were 0⋅3 ± 0⋅2 % and 0⋅3 ± 0⋅2 % of total energy intake in each trimester. The mean serum DHA and EPA levels were 158⋅9 ± 43⋅4 μg/mL and 181⋅9 ± 65⋅5 μg/mL in each trimester. There was no significant difference in the mean dietary DHA and EPA intakes between the second and third trimesters. There was no significant difference in participant characteristics between those who were included and excluded from this study.

**Table 1.** Participant characteristics

	Second trimester	Third trimester	
	mean ± sd or *n* (%)	mean ± sd or *n* (%)	*P*-value^a^
Age at delivery (years)	35⋅32 ± 4⋅31		
Pre-pregnancy BMI	21⋅36 ± 3⋅58		
Parity			
Primipara	77 (66⋅4)		
Multipara	39 (33⋅6)		
Education			
Junior high or high school	5 (4⋅3)		
Junior/technical college	21 (18⋅1)		
University	65 (56⋅0)		
Graduate school	25 (21⋅6)		
Family income (Japanese yen per year)			
<3 million	2 (1⋅7)		
3–<5 million	12 (10⋅3)		
5–<7 million	18 (15⋅5)		
7–<9 million	17 (14⋅7)		
≥9 million	67 (57⋅8)		
Working			
Yes	89 (76⋅7)	9 (7⋅8)	
No	27 (23⋅3)	107 (92⋅2)	
Using supplements containing DHA and/or EPA			
Every day	11 (9⋅5)	16 (13⋅8)	
5–6 times per week	0 (0⋅0)	2 (1⋅7)	
3–4 times per week	1 (0⋅9)	3 (2⋅6)	
1–2 times per week	1 (0⋅9)	2 (1⋅7)	
Sometimes	7 (6⋅0)	4 (3⋅4)	
Never	96 (82⋅8)	89 (76⋅7)	
Dietary DHA + EPA intakes (% of energy)	0⋅3 ± 0⋅2	0⋅3 ± 0⋅2	0⋅691
Serum DHA + EPA level (μg/mL)	158⋅9 ± 43⋅4	181⋅9 ± 65⋅5	<0⋅001*

BMI, body mass index; DHA, docosahexaenoic acid; EPA, eicosapentaenoic acid; sd, standard deviation.

a The paired Student's *t*-test was used.

* *P*-value < 0⋅01.

### Supplement use and serum DHA and EPA levels

Table 2 shows the medians (IQRs) of serum DHA and EPA levels of the three frequency of supplement use groups. There were no differences in the percentage intake of dietary DHA and EPA (second trimester: *P* = 0⋅508; third trimester: *P* = 0⋅968) among the three frequency of supplement use groups (data not shown in the table). The serum DHA and EPA levels of regular users were the highest in both the second and third trimesters. The results of Tukey's multiple comparison analysis showed significant differences in serum DHA and EPA levels between regular and never users in the second trimester.

**Table 2.** Serum DHA/EPA level medians by frequency of use of DHA/EPA supplements

	Regular^a^					Irregular^a^					Never^a^					Regular *v.* Irregular	Regular *v.* Never	Irregular *v.* Never
	*N*	median	IQR			*n*	median	IQR			*n*	median	IQR			*P*-value^b^	*P*-value^b^	*P*-value^b^
Serum DHA + EPA level (μg/mL)																		
Second trimester	11	185⋅6	166⋅1	−	202⋅4	9	149⋅6	117⋅8	−	171⋅0	96	146⋅1	127⋅6	−	177⋅5	0⋅106	0⋅027	0⋅966
Third trimester	18	199⋅3	160⋅1	−	250⋅5	9	153⋅8	115⋅9	−	197⋅2	89	172⋅6	137⋅3	−	205⋅8	0⋅138	0⋅078	0⋅808

a Definition of frequency: regular; ≥5 times per week. Irregular; sometimes – ≤4 times per week. Never; never.

b A Tukey's multiple comparison analysis was performed.

*P-value < 0⋅05.

DHA, docosahexaenoic acid; EPA, eicosapentaenoic acid; IQR, interquartile range.

### Dietary intake and serum DHA and EPA levels

Table 3 shows the medians (IQRs) of dietary DHA and EPA intakes by food group. It also shows the correlation coefficients between DHA and EPA intakes by food group and serum DHA and EPA levels in pregnant women who were not using supplements during the second trimester. The dietary intake of DHA and EPA from fish and shellfish was significantly correlated with serum DHA and EPA levels in both the second and third trimesters.

**Table 3.** The amount of DHA and EPA intakes by food group and correlation coefficients between serum DHA/EPA levels among pregnant women who were not using supplements

	Second trimester (*n* 96)						Third trimester (*n* 89)					
Dietary DHA + EPA intakes from	Median (mg/d)	IQR (mg/d)			*ρ*^a^	*P*-value	Median (mg/d)	IQR (mg/d)			*ρ*^a^	*P*-value
Fish and shellfish	322⋅5	160⋅2	−	586⋅0	0⋅30	0⋅003*	375⋅6	181⋅4	−	553⋅1	0⋅33	0⋅001*
Eggs	28⋅3	25⋅5	−	56⋅6	0⋅01	0⋅942	28⋅3	25⋅5	−	56⋅6	0⋅01	0⋅939
Sugar and confectionaries	8⋅3	3⋅3	−	16⋅3	0⋅06	0⋅592	8⋅3	3⋅4	−	16⋅6	0⋅05	0⋅635
Meat	6⋅0	3⋅8	−	9⋅1	0⋅07	0⋅487	6⋅0	3⋅8	−	10⋅2	0⋅14	0⋅178
Grain products	2⋅8	1⋅5	−	6⋅3	0⋅12	0⋅247	4⋅5	2⋅5	−	7⋅1	0⋅06	0⋅558
Dairy products	1⋅7	1⋅2	−	2⋅4	0⋅07	0⋅515	1⋅9	1⋅3	−	3⋅0	0⋅02	0⋅862
Vegetables other than green and yellow vegetables	1⋅5	0⋅7	−	3⋅7	0⋅15	0⋅142	1⋅5	0⋅7	−	3⋅7	0⋅07	0⋅483
All other foods^b^	0⋅0	0⋅0			−	−	0⋅0	0⋅0			−	−

a Spearman's correlation coefficient was used to determine the correlation between dietary DHA and EPA intakes from each food group and serum DHA and EPA levels.

b Including pulses, potatoes, fat and oil, fruit, and green and yellow vegetables.

* *P*-value < 0⋅05.

DHA, docosahexaenoic acid; EPA, eicosapentaenoic acid; IQR, interquartile range.

## Discussion

### Overview

The results of this study suggest that those who used DHA and/or EPA supplements ≥5 times per week had significantly higher serum DHA and EPA levels in both the second and third trimesters than those who never used supplements. However, there were no significant differences in the dietary intake of DHA and EPA between the three frequency of supplement use groups. Additionally, serum DHA and EPA levels were significantly correlated with DHA and EPA intake from fish and shellfish among participants who were not using DHA and/or EPA supplements during both the second and third trimesters.

### Serum DHA and EPA levels compared to other study populations

In this study, the mean serum DHA and EPA levels were higher than reported in previous studies that also analysed serum DHA and EPA levels.^(11,12)^ This may reflect the characteristics of the study population. First, the number of supplement users may have influenced the results. According to previous studies, only a small number of pregnant women used supplements containing DHA or fish oil^(11,12)^ compared with the present study, in which DHA and/or EPA supplement users constituted approximately 9–14 % of the study's participants. Second, demographic characteristics, such as maternal age, educational levels, smoking habits, and alcohol consumption, may have affected the results. Compared with previous studies, the mean maternal age and the proportion of women with a high educational level were higher.^(11,12)^ Maternal age and maternal educational levels are known to be positively associated with maternal DHA and EPA levels.^(9,11,12)^ In contrast, smoking and alcohol consumption are known to be negatively associated with maternal DHA and EPA levels. Since none of the participants in this study currently smoked or consumed alcohol, the mean DHA and EPA levels were likely to be higher (see Supplementary Tables 1 and 2). In the present Japanese study, fish and shellfish consumption was 30⋅2 and 29⋅3 g/4184000 J in the second and third trimesters, respectively. However, due to differences in the unit of measurement and the food assessment methods, it is difficult to compare fish and shellfish consumption in the present study to that of previous studies. For instance, one study in Spain reported fish and seafood consumption among pregnant women in grams per day^(12)^ compared with grams per 4184000 J used in the present study. Another study reported fish consumption among pregnant women in Brazil by percentage of energy.^(11)^

Since serum samples reflect only short-term intake, serum DHA and EPA levels should be interpreted as approximate levels for the previous month. It is more appropriate to measure DHA and EPA levels of the erythrocyte membranes, which is considered the gold standard because these levels reflect long-term intakes. However, by using BDHQ to determine dietary intakes during the previous month, only a relatively short period is accounted for.

### Supplement use and biomarkers

Of the 116 pregnant women, 11 (9⋅5 %) in the second trimester and 18 (15⋅5 %) in the third trimester were using supplements ≥5 times per week. A nationwide study conducted in Japan between 2011 and 2014 showed that 2⋅57 % of pregnant women were using DHA and/or EPA supplements.^(1)^ The number of pregnant women using DHA and/or EPA supplements in the present study was higher than in previous studies. The number of pregnant women using DHA and/or EPA supplements was likely to have increased over time. DHA and/or EPA supplements are marketed as supplements for pregnant women, and most of them are displayed near folic acid supplements, which are recommended for pre-pregnancy use in Japan. Additionally, some DHA and/or EPA supplements contain a combination of other nutrients, such as folic acid, iron, calcium, and some vitamins. Moreover, the number of pregnant women using folic acid supplements is increasing yearly. The percentage of pregnant women using adequate quantities of folic acid supplements increased from 7⋅4 to 9⋅1 % between 2011 and 2014^(25)^ and 18 % from 2013 to 2017.^(26)^ It is possible that pregnant women have become more aware of supplement use and that some have chosen to use supplements containing DHA and/or EPA.

In addition, compared with previous studies, our study participants were older and had higher educational levels, which are known to be associated with DHA and/or EPA supplement use.^(27–29)^ These factors also appear to be associated with DHA and/or EPA levels. However, due to the small sample size, we were unable to adjust for these factors (Supplementary Table 1).

As shown in Table 2, the women who used DHA and/or EPA supplements ≥5 times per week had higher serum DHA and EPA levels. In addition, there was no significant difference in dietary DHA and EPA intakes between those who used the supplements ≥5 times per week and those who did not. These results may imply that the use of these supplements has positive effects on serum DHA and EPA levels. Some studies have suggested that the presence of larger amounts of DHA and/or EPA in supplements was associated with higher maternal DHA and EPA levels.^(30,31)^ However, pregnant women use various supplements containing different amounts of DHA and/or EPA. It should be noted that the multiple comparison analysis revealed significant differences in median serum DHA and EPA levels only between regular and never users. There were no significant differences in median serum DHA and EPA levels between irregular and never users. Therefore, regular use of DHA and/or EPA supplements may have an effect on increasing maternal blood DHA and EPA levels.

An additional analysis using the Student's *t*-test showed that there were no significant differences in the gestational weeks at delivery or in birth weight between participants who used DHA and/or EPA supplements ≥5 times per week and those who used them ≤4 times per week (data were not shown in the table). However, given the sample size in the current study, we were unable to undertake analysis to examine associations with preterm birth. In order to examine the association between DHA and/or EPA and preterm birth, other factors associated with preterm birth need to be carefully adjusted for.

### Dietary intakes and biomarkers

Fish and shellfish are regarded as abundant sources of DHA and EPA. Previous studies have revealed the correlation between the consumption of seafood and the levels of serum DHA and EPA level.^(11,12)^ This study not only examined the impact of fish and shellfish but also encompassed a comprehensive assessment of all dietary sources of DHA and EPA, employing a validated tool for dietary evaluation. Consequently, the levels of DHA and EPA in the serum exhibited a significant association with the intake of these fatty acids from fish and shellfish during the second and third trimesters, focusing on participants who did not incorporate DHA and/or EPA supplements. Therefore, it can be inferred that fish and shellfish exert more influence on the serum levels of DHA and EPA compared to alternative food sources.

Typically, the second trimester holds great importance for pregnant women, as many can regulate their dietary patterns without the presence of nausea and vomiting, which are frequently experienced during the first trimester. If a higher intake of dietary DHA and EPA plays a role in the prevention of depressive symptoms or preterm births, it is reasonable to assume that an augmented consumption of DHA and EPA from fish and shellfish could be effective, given its capacity to elevate maternal serum levels of these fatty acids during the second and third trimesters.

### Limitations

This study had some limitations. First, ‘supplements’ were not clearly defined, and the frequency of use was self-reported. Some pregnant women may have reported not using products containing DHA and/or EPA, while unknowingly using them. Brand names of supplements or the amount of DHA and/or EPA were not recorded; hence, the absolute DHA and/or EPA intakes from supplements were not determined properly. Second, the findings of this study should be cautiously generalised to other populations. Due to the older age and higher educational levels of the participants in this study compared with a previous nationwide study, this study's participants’ serum DHA and EPA levels may have been higher than those of the general population of pregnant women in Japan.^(1)^ It could not be adjusted because of the small sample size. Therefore, the results of this study may underestimate the difference in serum DHA and EPA levels between irregular and never users of these supplements. We conducted additional analysis to investigate potential sample bias caused by the high drop-out rate. The outcomes of these analyses revealed a statistically significant discrepancy, with a notably higher proportion of multiparous individuals observed among the participants who dropped out from the study in comparison to the 116 participants who completed all the questionnaires (*χ*^2^ = 4⋅11, *P* = 0⋅04). While we were unable to directly assess their specific circumstances, it is plausible that pregnant women who lacked the time or mental capacity to participate in the research may embrace distinct interests or dietary habits concerning food and supplements.

In conclusion, this was the first study to investigate serum DHA and EPA levels in relation to both dietary intakes and supplement use among pregnant women in Japan. This study revealed that both greater DHA and EPA intake from fish and shellfish and using supplements ≥5 times per week were associated with higher serum DHA and EPA levels in a population that was considered to have high blood DHA and EPA levels. Further studies are necessary to determine the association between serum DHA and/or EPA levels and the risk of gestational outcomes, such as preterm birth or depression symptoms stratified by maternal risk. This study is likely to help with interpreting the findings of future studies conducted among a similar population.

## Abbreviated names

Wakabayashi N, Haruna M, Yonezawa K, Sasagawa E, Usui Y, Ohori R, Aoyama S, Sasaki S, Nagamatsu T.

## Supporting information

Wakabayashi et al. supplementary material Wakabayashi et al. supplementary material

## Supplementary material

The supplementary material for this article can be found at https://doi.org/10.1017/jns.2023.105
