# Supplementary material for: Association of serum docosahexaenoic acid and eicosapentaenoic acid levels with dietary intakes and supplement use during pregnancy: a prospective observational study
Source: J Nutr Sci. 2023 Dec 14;12:e125. doi: 10.1017/jns.2023.105 (PMC10753464; doi:10.1017/jns.2023.105)
Supplement: Wakabayashi et al. supplementary material [file S2048679023001052sup001.docx]

**Supplementary table1.** Mean age, education level, and pre-pregnancy smoking status for the three strata of supplementation

|  |  | Strata of DHA/EPA supplements | | | | | | | | | | | |
| --- | --- | --- | --- | --- | --- | --- | --- | --- | --- | --- | --- | --- | --- |
|  |  | Second trimester | | | | | | Third trimester | | | | | |
|  |  | Regular^†^ | | Irregular^†^ | | Never^†^ | | Regular^†^ | | Irregular^†^ | | Never^†^ | |
| Mean age (years) | | 38.4±4.5 | | 36.7±4.2 | | 34.8±4.1 | | 36.6±5.0 | | 35.8±3.4 | | 35.0±4.2 | |
| Education level (n (%)) | |  | |  | |  | |  | |  | |  | |
|  | Junior high or high school | 1 | (20.0) | 1 | (20.0) | 3 | (60.0) | 1 | (20.0) | 1 | (20.0) | 3 | (60.0) |
|  | Junior/technical college | 2 | (9.5) | 3 | (14.3) | 16 | (76.2) | 3 | (14.3) | 2 | (9.5) | 16 | (76.2) |
|  | University | 5 | (7.7) | 5 | (7.7) | 55 | (84.6) | 10 | (15.4) | 6 | (9.2) | 49 | (75.4) |
|  | Graduate school | 3 | (12.0) | 0 | (0.0) | 22 | (88.0) | 4 | (4.5) | 0 | (0.0) | 84 | (95.5) |
| Smoking (n (%)) | | 0 | (0.0) | 0 | (0.0) | 0 | (0.0) | 0 | (0.0) | 0 | (0.0) | 0 | (0.0) |

† Definition of frequency: Regular; ≥5 times per week. Irregular; sometimes – ≤4 times per week. Never; Never.

**Supplementary table2.** Mean serum DHA+EPA levels by strata of age and education levels

|  |  |  | Mean serum DHA+EPA levels (µg/mL) | |
| --- | --- | --- | --- | --- |
| Age (years) | | (n) | Second trimester | Third trimester |
|  | -24 | 1 | 102.1 | 119.8 |
|  | 25-29 | 6 | 153.3 | 155.2 |
|  | 30-34 | 44 | 147.9 | 168.2 |
|  | 35-39 | 44 | 164.1 | 196.7 |
|  | 40-44 | 20 | 175.0 | 190.2 |
|  | 45-49 | 1 | 185.4 | 188.9 |
| Education levels | |  |  |  |
|  | Junior high or high school | 5 | 135.9 | 137.1 |
|  | Junior/technical college | 21 | 161.8 | 185.2 |
|  | University | 65 | 158.8 | 179.8 |
|  | Graduate school | 25 | 161.4 | 193.6 |
